# Supplementary material for: Dynamically Tunable Assemblies of Superparamagnetic Nanoparticles Stabilized with Liquid Crystal-like Ligands in Organic Thin Films
Source: Nanomaterials (Basel). 2023 Nov 6;13(21):2908. doi: 10.3390/nano13212908 (PMC10648093; doi:10.3390/nano13212908)
Supplement: Supplementary file 1 [file nanomaterials-13-02908-s001.zip › nanomaterials-2591860-supplementary.pdf]

Supporting Information

Supplementary Note S1: Synthesis of LC-like ligand L

The synthetic pathway was similar to that described in our previous article[1] until the compound denoted as (A) was obtained.

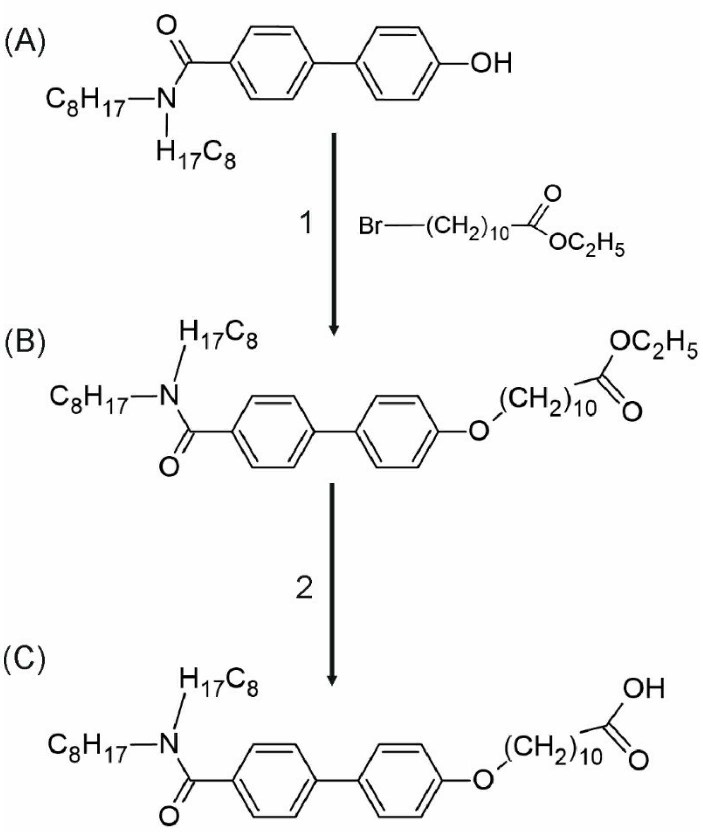

Figure S1. The synthetic path of ligand L. Reagents and conditions: 1. Br(CH)$_2$COOEt, RT; 2. KOH, THF, RT.

To a solution of 9.2 g (17 mmol) of compound (A) in 300 mL of DMF, 2.82 g (20.4 mmol) K_2CO_3, 3.39 g (20.4 mmol) KI was added. Then 6.27 g (20.4 mmol) of ethyl 12-bromododecanoate was added. The reaction was carried out at 90 °C under reflux for 8 hours, controlling its progress by thin layer chromatography. The cooled to room temperature contents of the flask was poured into a beaker containing a mixture of water and crushed ice. The resulting precipitate was filtered and left to air dry. The crude product (B) was purified by column chromatography using toluene/chloroform in 7/3 volume ratio as the eluent. 9.77 g (12.58 mmol) of compound (B) as a white solid was obtained. The yield of the reaction was 74%.

Supplementary Note S2a: Estimation of the volume of the unit cell

Namely, if the first peak was fitted, the none of the consecutive modelled reflections matched the second experimental peak. We next decided to use the P63/mmc space group, also often found for packing of spheres. In this case we were able to find local minima of fits, giving a relatively wide range of unit cells with sizes a ~ 8.3 nm, c ~ 15.5 nm to a ~ 9.0 nm, c ~ 10.3 n

m, translating to the unit cell volumes from ~ 718 to ~ 934 nm^3. Since each unit cell is composed of two particles, the single particle volume would be from 369 to 467 nm^3 (there are two particles forming a single unit cell).

Supplementary Note S**2b:** Estimation of the volume of a single HB

It is important to estimate a volume of a single HB particle based on the experimental data. This task is not trivial simply using the size of the metal oxide core and sizes of the ligands, as we know that particles covered with promesogenic ligands can change their volume depending on the temperature (namely, growing entropic requirements of ligands with raising the temperature above phase transitions of the ligands), we do not know how much the ligands from neighbor particles interdigitate, and since the ligands are immobilized on a curved surface, the density of the organic material may vary from the regular bulk values.

Supplementary Figures

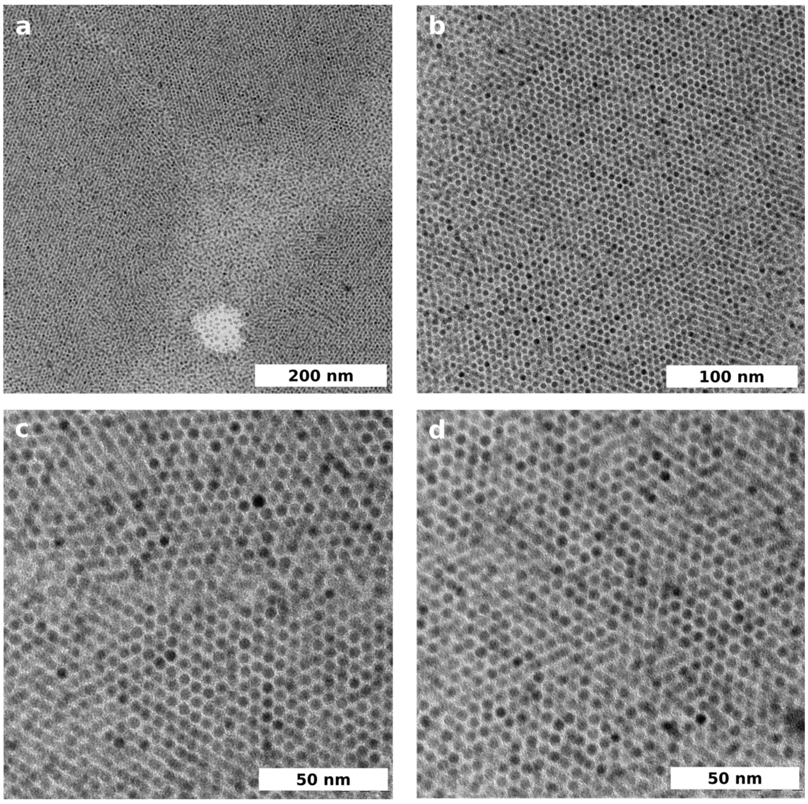

Figure S2. TEM pictures of nanoparticles HB.

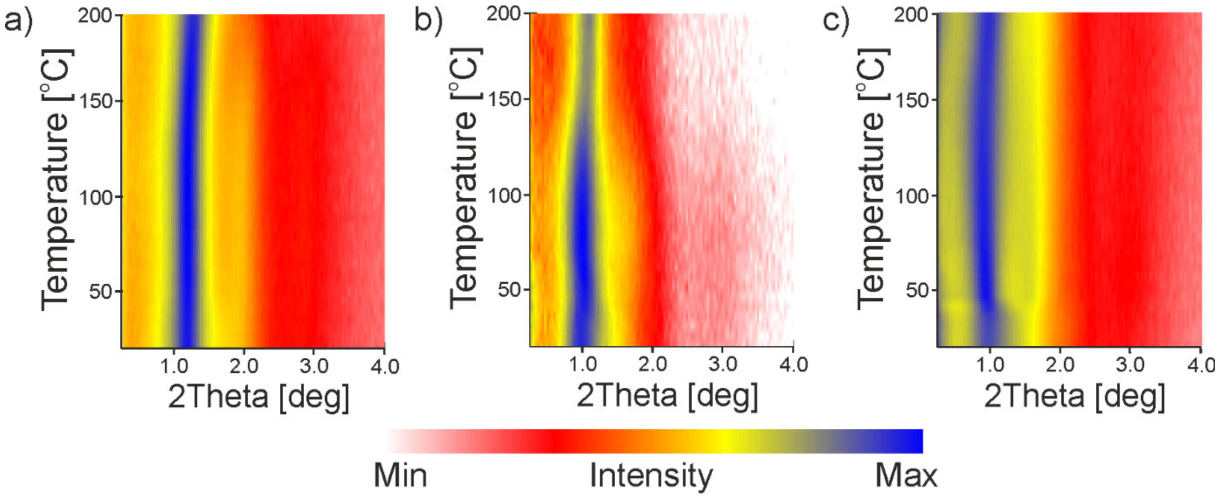

Figure S3. Temperature evolution of an SAXRD pattern obtained by the heating for a) EA1, b) EA2, and c) EA3 composites.

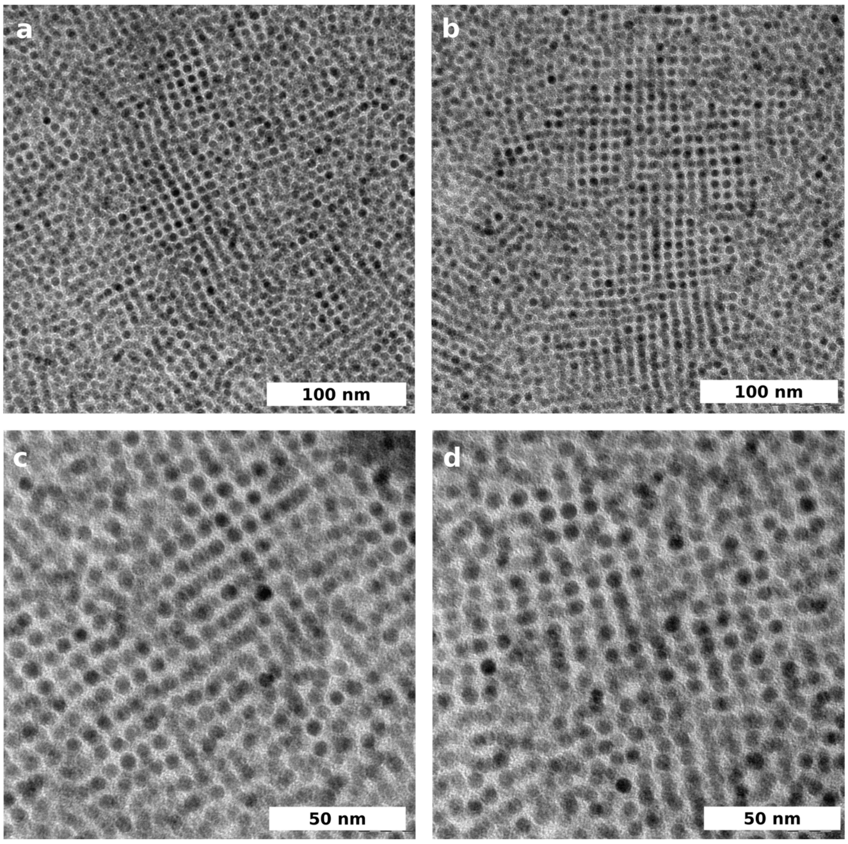

Figure S4 TEM pictures of nanoparticles EB2.

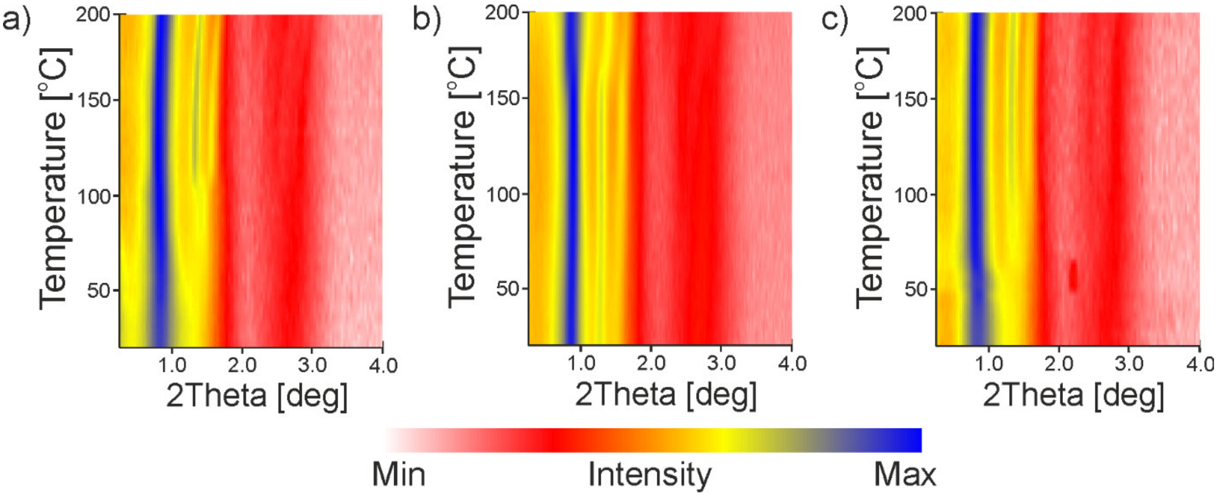

Figure S5. Temperature evolution of an SAXRD pattern obtained by the heating for a) EB1, b) EB2, and c) EB3 composites.

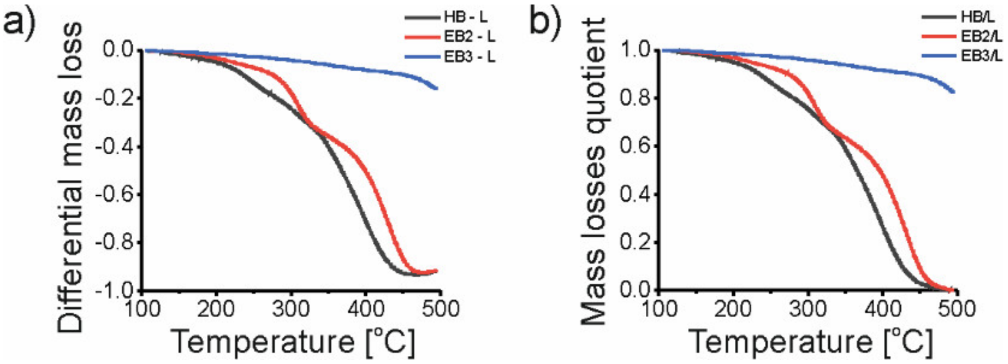

Figure S6. Additional analysis of thermogravimetric data.

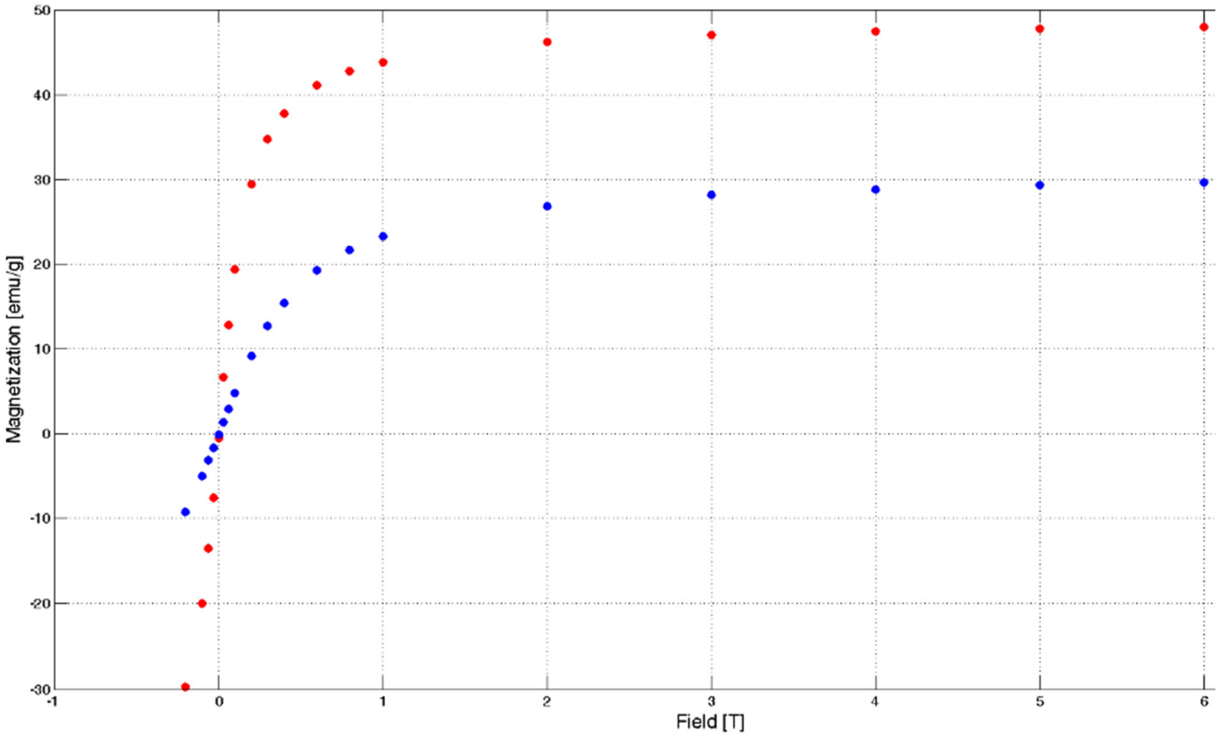

Figure S7. Magnetization as a function of magnetic field at 350K for IONPs of Serie A (red) and EA2 (blue).

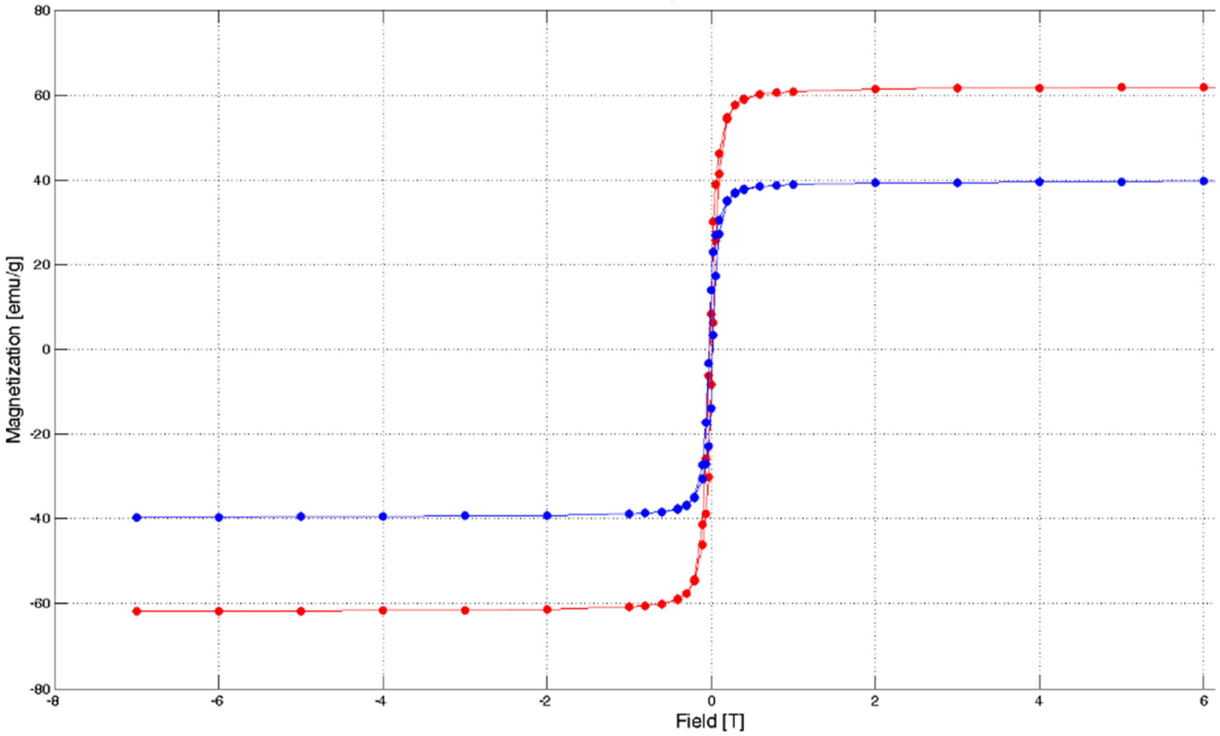

Figure S8. Full range of magnetization hysteresis for Series A (red) and EA2 (blue) samples at T=2K.

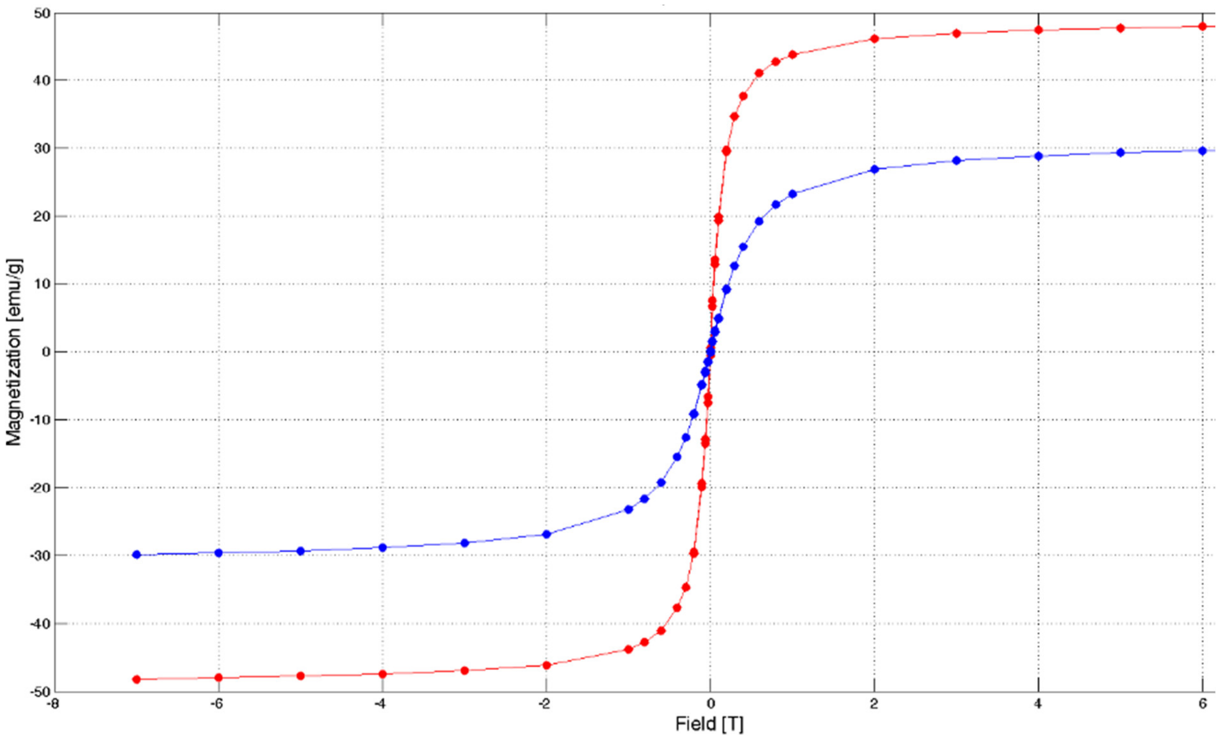

Figure S9. Hysteresis for samples of Serie A (red) and EA2 (blue) at 350K.

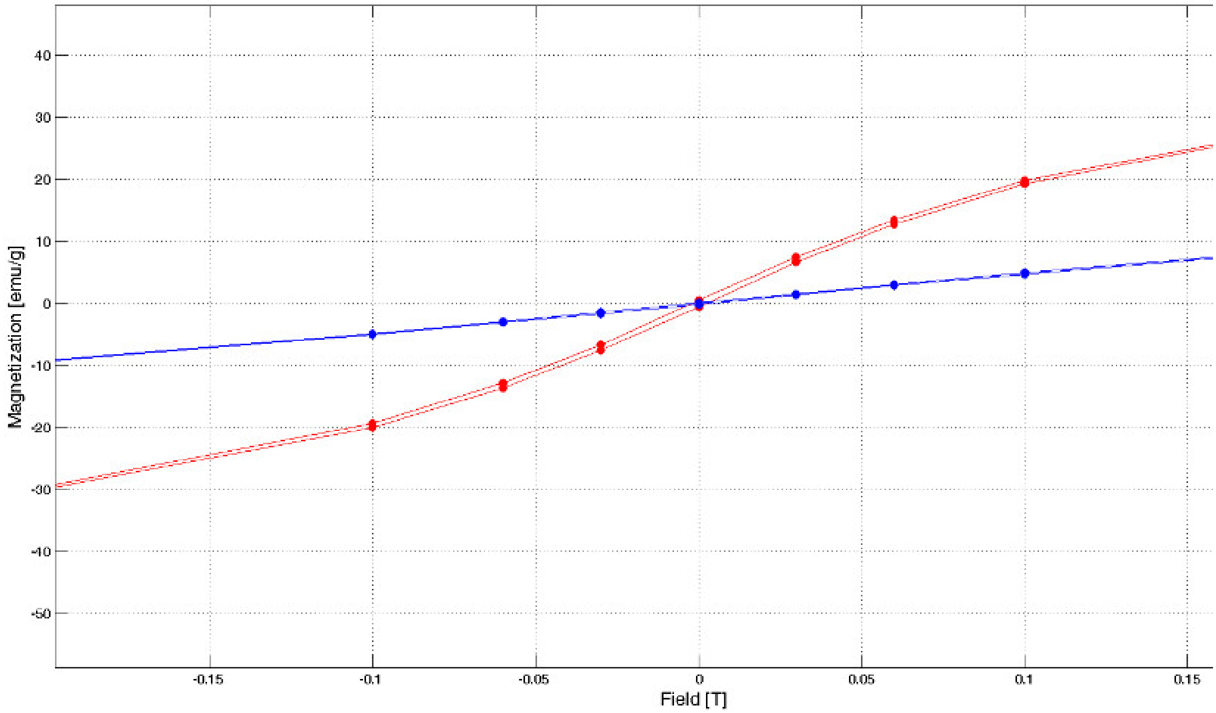

Figure S10. Hysteresis for samples of Serie A (red) and EA2 (blue) at 350K for magnetic field in range -0.15-0.15 T.

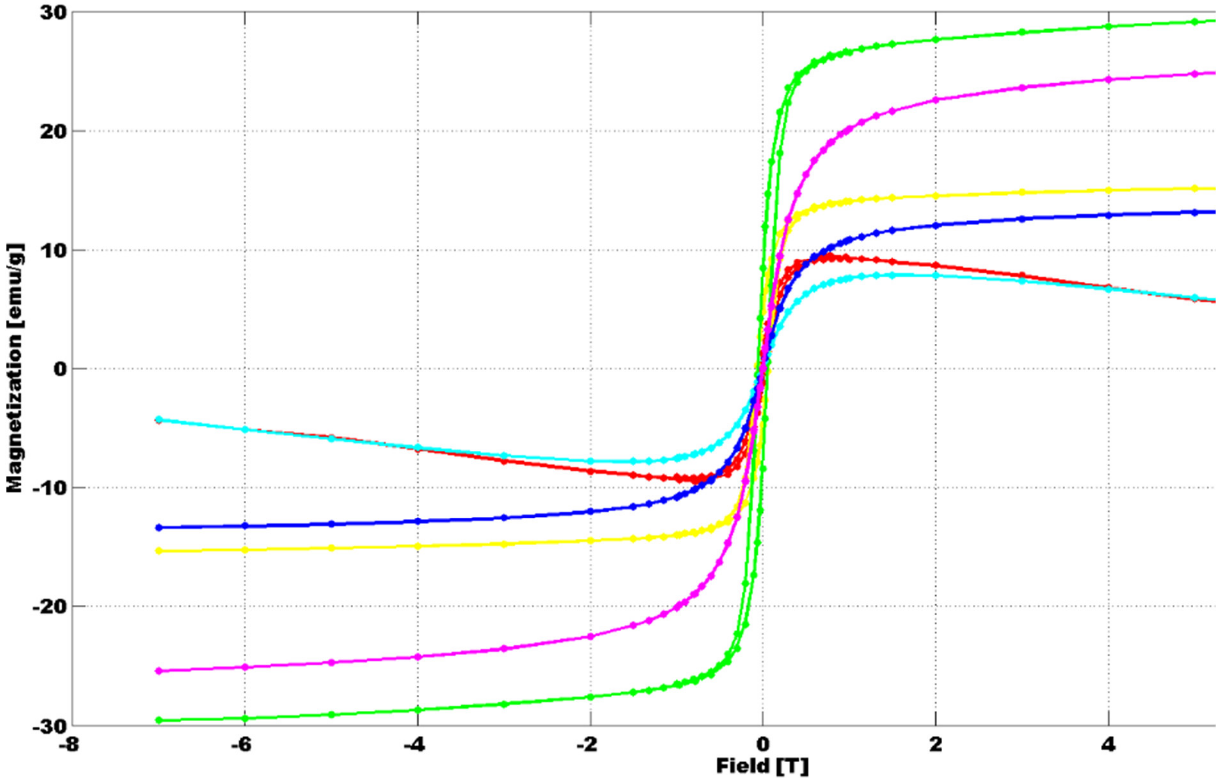

Figure S11. The graph of the dependence of magnetization on the magnetic field for three samples at 2K: IONPs Serie B (red), EB2 (yellow), and HB (green) and at 300K: IONPs Serie B (cyan), EB2 (blue), and HB (purple).

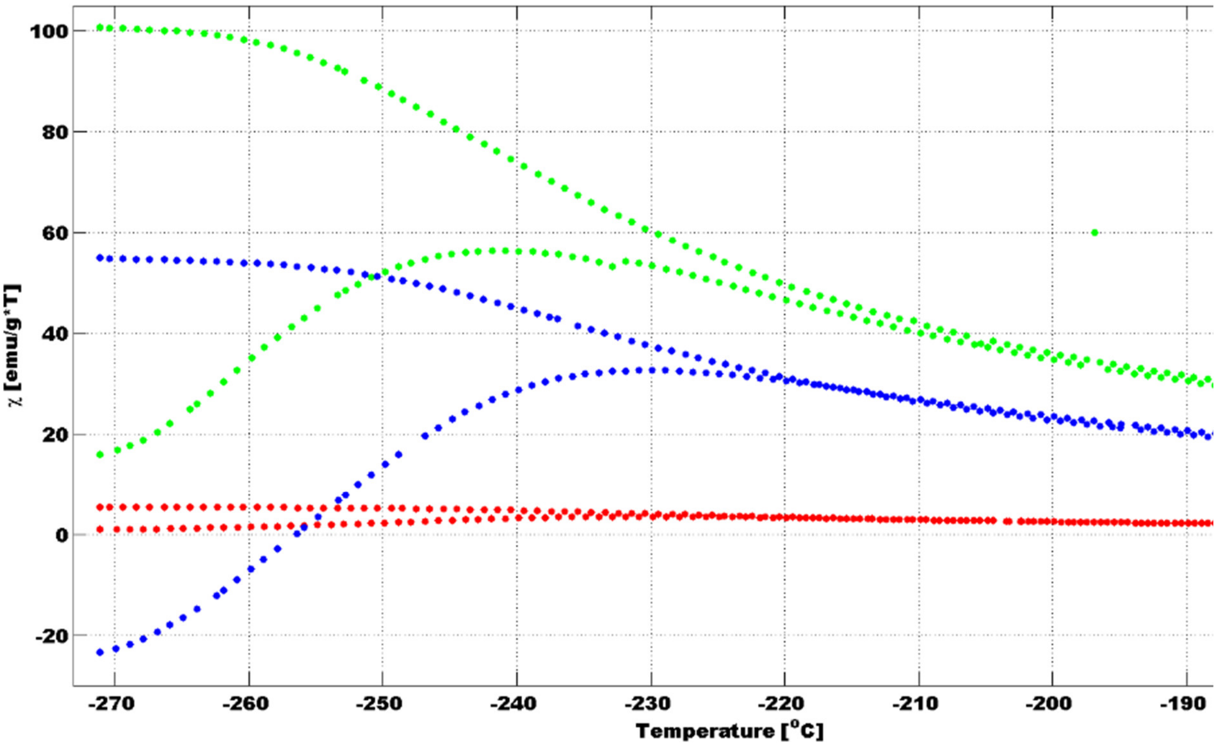

Figure S12. The graph of the dependence of magnetic susceptibility on temperature for: IONPs Serie B, EB2, HB (red, green and blue, respectively) for temperatures ranging from -272 to -190 °C.

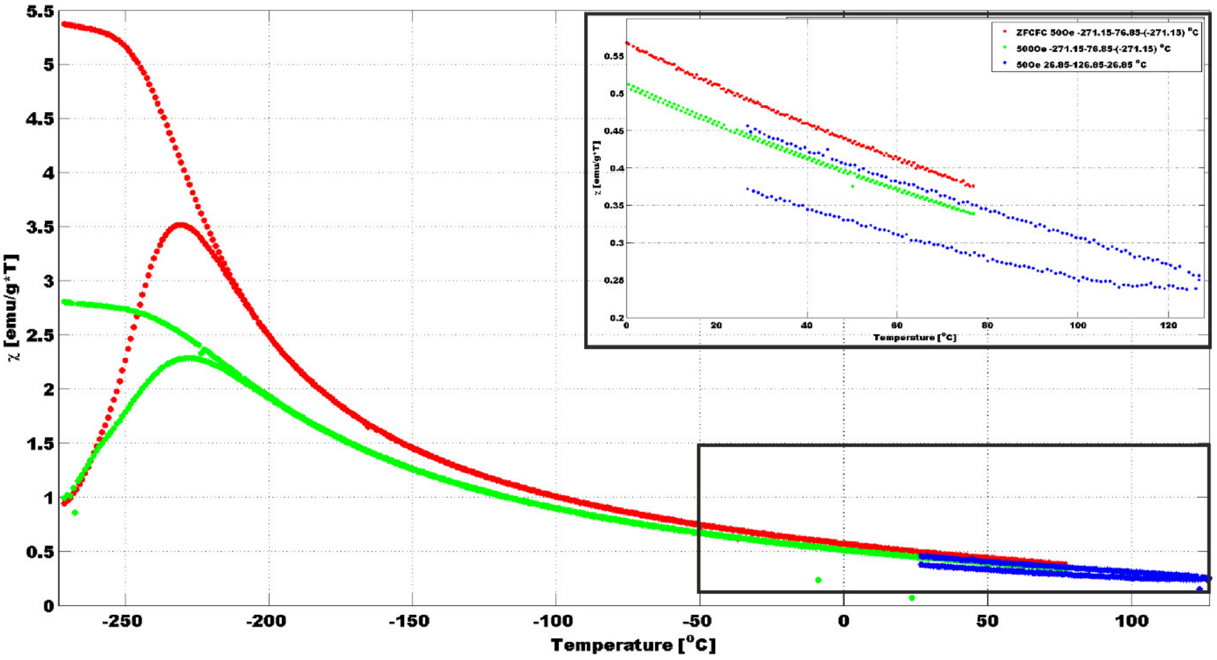

Figure S13. The graph of the dependence of magnetic susceptibility on temperature for: IONPs Serie B, EB2, HB (red, green and blue, respectively).

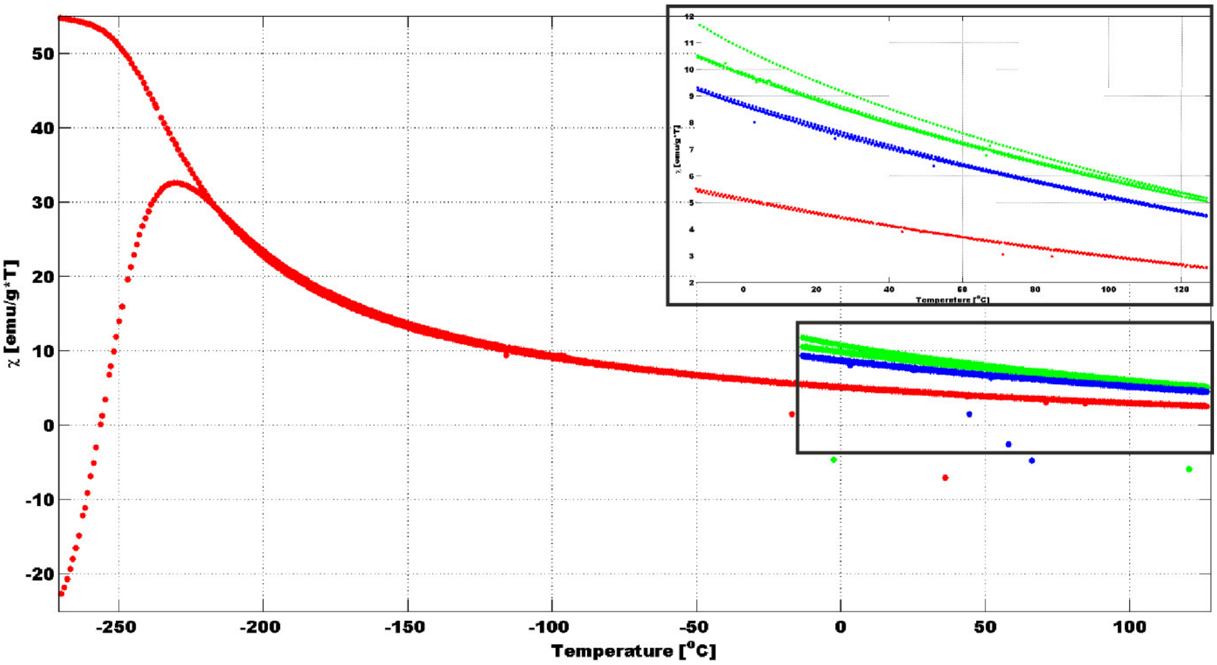

Figure S14. The graph of the dependence of magnetic susceptibility on temperature for: IONPs Serie B, EB2, HB (red, green and blue, respectively).

Supplementary Tables

Table S1. The chemical composition based on XPS.

	Element	Mass percentage [%]
OA	O	11.30
	C	76.60
Fe_3O_4	O	27.20
	Fe	72.70
Ligand L	O	10.30
	N	1.80
	C	78.30
Primary IONPs (Serie B)	Fe	7.69
	O	32.95
	C	59.36
EB2	Fe	0.00
	O	18.79
	N	1.20
	C	80.01
HB	Fe	1.15
	O	29.01
	N	1.70
	C	68.14

Literature

1. Temperature-controlled liquid crystalline polymorphism of gold nanoparticles, Soft Matter, 2011,7, 10561-10564
